# Mutations in *CPAMD8* Cause a Unique Form of Autosomal-Recessive Anterior Segment Dysgenesis

**DOI:** 10.1016/j.ajhg.2016.09.022

**Published:** 2016-11-10

**Authors:** Sek-Shir Cheong, Lisa Hentschel, Alice E. Davidson, Dianne Gerrelli, Rebecca Davie, Roberta Rizzo, Nikolas Pontikos, Vincent Plagnol, Anthony T. Moore, Jane C. Sowden, Michel Michaelides, Martin Snead, Stephen J. Tuft, Alison J. Hardcastle

**Affiliations:** 1 Institute of Ophthalmology, University College London, London EC1V 9EL, UK; 2 Great Ormond Street Institute of Child Health, University College London, London WC1N 1EH, UK; 3 Vitreoretinal Research Group, Cambridge University National Health Service Foundation Trust, Cambridge CB2 0QQ, UK; 4 Moorfields Eye Hospital, London EC1V 2PD, UK; 5 Genetics Institute, University College London, London WC1E 6BT, UK; 6 Ophthalmology Department, School of Medicine, University of California, San Francisco, San Francisco, CA 94143, USA

**Keywords:** anterior segment dysgenesis, CPAMD8, iris, lens, development, eye, A2M/C3, WES

## Abstract

Anterior segment dysgeneses (ASDs) comprise a spectrum of developmental disorders affecting the anterior segment of the eye. Here, we describe three unrelated families affected by a previously unclassified form of ASD. Shared ocular manifestations include bilateral iris hypoplasia, ectopia lentis, corectopia, ectropion uveae, and cataracts. Whole-exome sequencing and targeted Sanger sequencing identified mutations in *CPAMD8* (C3 and PZP-like alpha-2-macroglobulin domain-containing protein 8) as the cause of recessive ASD in all three families. A homozygous missense mutation in the evolutionarily conserved alpha-2-macroglobulin (A2M) domain of CPAMD8, c.4351T>C (p. Ser1451Pro), was identified in family 1. In family 2, compound heterozygous frameshift, c.2352_2353insC (p.Arg785Glnfs^∗^23), and splice-site, c.4549-1G>A, mutations were identified. Two affected siblings in the third family were compound heterozygous for splice-site mutations c.700+1G>T and c.4002+1G>A. *CPAMD8* splice-site mutations caused aberrant pre-mRNA splicing in vivo or in vitro. Intriguingly, our phylogenetic analysis revealed rodent lineage-specific *CPAMD8* deletion, precluding a developmental expression study in mice. We therefore investigated the spatiotemporal expression of *CPAMD8* in the developing human eye. RT-PCR and in situ hybridization revealed *CPAMD8* expression in the lens, iris, cornea, and retina early in development, including strong expression in the distal tips of the retinal neuroepithelium that form the iris and ciliary body, thus correlating *CPAMD8* expression with the affected tissues. Our study delineates a unique form of recessive ASD and defines a role for CPAMD8, a protein of unknown function, in anterior segment development, implying another pathway for the pathogenicity of ASD.

## Main Text

Anterior segment dysgeneses (ASDs) are a heterogeneous group of developmental conditions affecting the anterior segment of the eye, including the cornea, iris, lens, trabecular meshwork, and Schlemm’s canal. The trabecular meshwork and Schlemm’s canal regulate aqueous humor (AH) flow from the anterior chamber, which, when dysregulated, can lead to an increase in intraocular pressure (IOP).^1^, ^2^, ^3^ Elevated IOP is a major risk factor for the development of glaucoma, and approximately 50% of individuals with ASD experience visual loss from glaucoma.^1^, ^2^, ^3^ The clinical features of ASD include iris hypoplasia, an enlarged or reduced corneal diameter, corneal vascularization and opacity, posterior embryotoxon, corectopia, polycoria, an abnormal iridocorneal angle, ectopia lentis, and anterior synechiae between the iris and posterior corneal surface. The combinations of these features permit ASD classification; however, ASD displays extensive phenotypic and genotypic heterogeneity with overlapping clinical presentations.^1^, ^2^, ^3^ For example, individuals with Axenfeld-Rieger syndrome (ARS) typically have iris hypoplasia, posterior embryotoxon, anterior synechiae, corectopia, and polycoria with a high risk of secondary glaucoma, as well as dental, cardiac, and neurological anomalies. Although ARS is most commonly caused by heterozygous mutations in *FOXC1* (forkhead box C1) (MIM: 601090) and *PITX2* (pituitary homeobox 2) (MIM: 601542),^4^, ^5^, ^6^, ^7^ *FOXC1* and *PITX2* have also been associated with Peters anomaly (MIM: 604229)^5^, ^8^ and some cases of aniridia (MIM: 106210).^9^ However, most cases of aniridia are caused by heterozygous loss-of-function (LOF) mutations in *PAX6* (paired box protein 6) (MIM: 607108),^10^, ^11^ resulting in partial or complete iris hypoplasia, foveal hypoplasia, cataracts, and corneal opacification. Other genes that have been associated with ASD include *PITX3* (MIM: 602669), *FOXE3* (MIM: 601094), *BMP4* (MIM: 112262), *CHRDL1* (MIM: 300350), *LTBP2* (MIM: 602091), and *CYP1B1* (MIM: 601771).^1^, ^2^, ^3^, ^12^, ^13^, ^14^ The majority of ASD-associated genes encode transcription factors, and others encode extracellular matrix proteins, BMP signaling pathway proteins, or glycosylating proteins, all of which play a crucial role in ocular development.^1^, ^2^, ^3^

**Figure 1:**
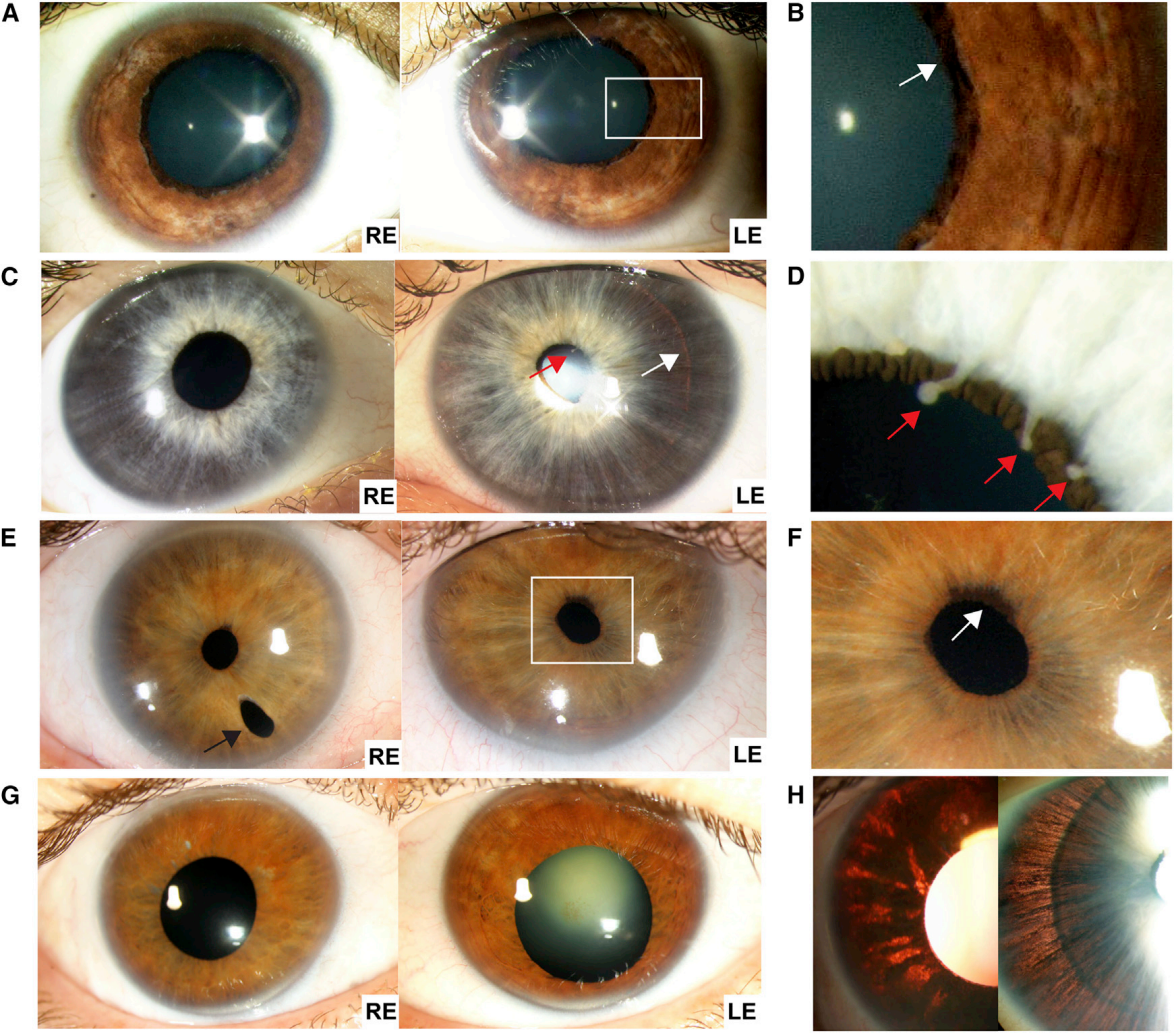
Clinical Images of the *CPAMD8*-Associated Anterior Segment Dysgenesis Phenotype (A and B) Proband from family 1, after pupil dilation. The pupils are displaced nasally, and the irides are hypoplastic with focal areas of iris pigment loss. There is ectropion uveae (arrow). (C and D) Proband from family 2. The pupils are displaced nasally, and the irides are thin and atrophic. The outline of the lens can be seen through the iris on retroillumination (white arrow). There are ectropion uveae and finger-like remnants arising from the collarette, suggestive of persistent pupillary membrane (red arrows). (E and F) Individual II:2 of family 3, showing temporally displaced pupils and mild ectropion uveae (white arrow). In the right eye there is an inferior surgical iridotomy (black arrow). (G) Individual II:3 of family 3. The pupils have been dilated and are displaced temporally. The irides are atrophic. The right eye has had cataract surgery and there is nuclear sclerosis of the left lens. (H) Images showing marked iris transillumination with loss of the iris pigment epithelium in individual II:3 of family 3 (left) and the proband from family 2 (right). RE, right eye; LE, left eye.

In this study, we describe four affected individuals from three unrelated families who had an unusual phenotype that did not fit with any of the previously described ASD criteria. All affected individuals shared predominant iris and lens abnormalities, including iris hypoplasia, iris transillumination defects, ectropion uveae, corectopia, iridodonesis with ectopia lentis, and cataracts. No retinal abnormalities or extra-ocular phenotypes were observed in our cohort (Figure 1 and Table 1). The anterior chamber angle was abnormal in one of the three families, but other phenotypes associated with ARS, such as posterior embryotoxon and extra-ocular phenotypes, were absent. Corneal opacity was absent and the fovea was not affected, distinguishing the condition of these four individuals from *PAX*6-associated disorders, which are characterized by reduced vision from foveal hypoplasia and corneal epithelial stem cell failure. One of the affected individuals with an abnormal anterior chamber angle developed increased IOP at the age of 49 years, which was likely exacerbated by lens dislocation and retinal detachment surgery. Other individuals (ranging from 17–50 years of age) had normal IOP (12–18 mmHg), thus distinguishing our cohort from individuals with mutations in *FOXC1, PITX2*, *LTBP2*, or *CYP1B1*, who frequently present with congenital or juvenile-onset increased IOP and glaucoma. The bilateral ocular manifestation of affected individuals also differs from ocular coloboma, which is caused by an aberrant closure of the optic fissure, resulting in a gap in ocular tissues, either unilaterally or bilaterally.^15^ The clinical features of affected individuals in this study are summarized in Table 1. All investigations were conducted in accordance with the principles of the Declaration of Helsinki and the study was approved by the local research ethics committee. Informed consent, including permission to publish photographs, was obtained from all participating individuals. Affected individuals and their relatives were clinically assessed by experienced ophthalmologists. Standard evaluation consisted of detailed ophthalmic examination and the additional measurement of the axial length of the eye and imaging of the anterior segment of the eye performed with ocular coherence tomography ([OCT] Visante, Carl Zeiss Meditec), b-scan ultrasonography, and optical interferometry (IOLMaster, Carl Zeiss Meditec).

**Figure 2:**
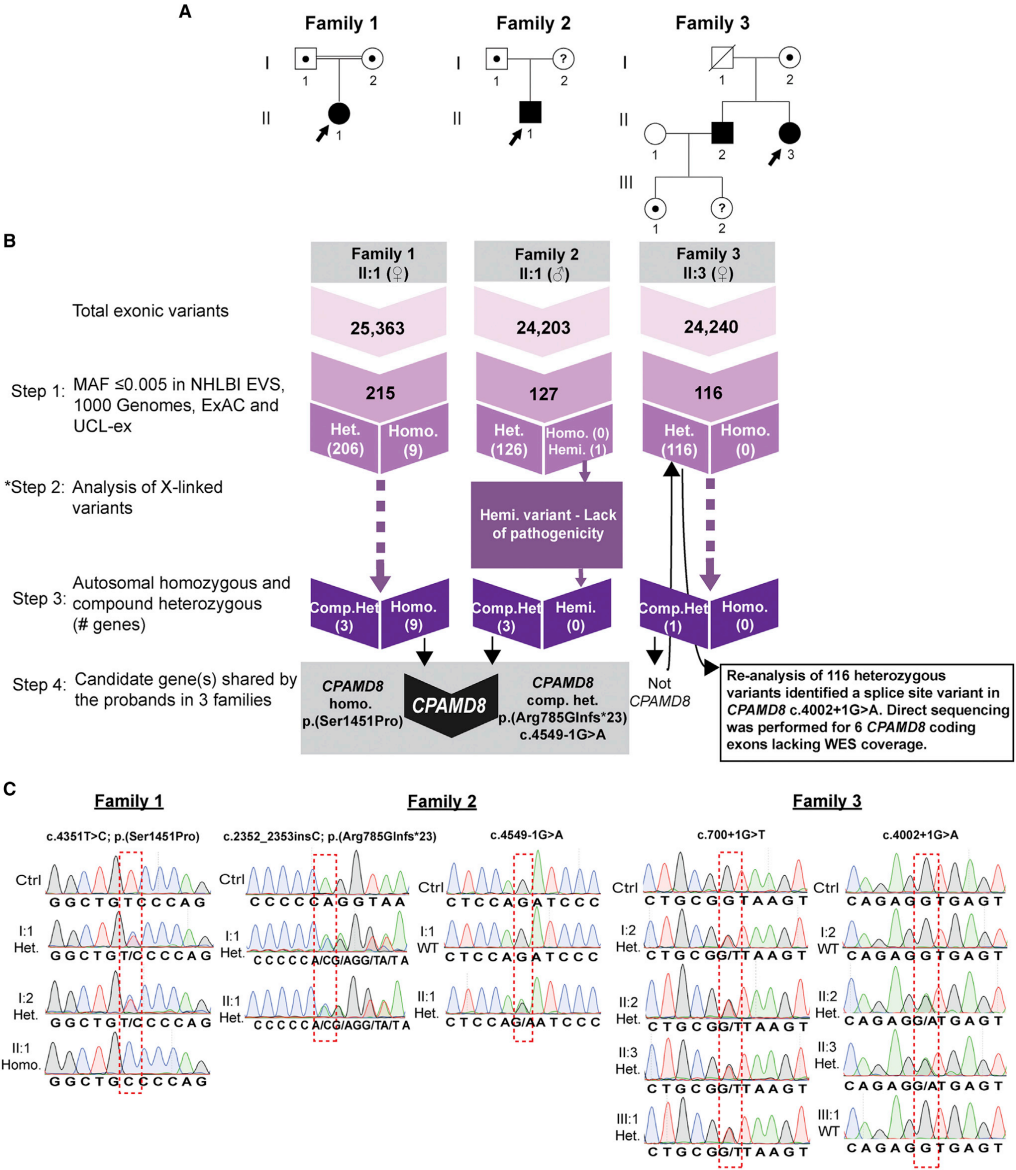
Identification of *CPAMD8* Mutations in Families Affected by Autosomal-Recessive ASD (A) Pedigrees of families 1, 2, and 3. An arrowhead indicates the proband in each family. A question mark indicates that DNA samples were not available for testing. (B) Flow chart showing the WES variant filtering strategies used in this study. Numbers in step 1 and 2 denote the number of variants. Step 1 selected for rare variants with a MAF ≤ 0.005 in 6500 NHLBI EVS, 1000 Genomes, the ExAC database, and our internal UCL-ex database. Variants were segregated according to zygosity status. Step 2 (applied to the proband from family 2 only) analyzed X-linked variants from the WES data. Step 3 selected for potential autosomal compound heterozygous and homozygous variants. Step 4 detected rare autosomal compound heterozygous or homozygous variants in gene(s) shared by the three probands. This step revealed a homozygous variant, c.4351T>C (p.Ser1451Pro), in family 1 and compound heterozygous variants, c.2352_2353insC (p.Arg785Glnfs^∗^23) and c.4549-1G>A, in *CPAMD8* in family 2. In family 3, re-analysis of rare heterozygous variants (step 1) led to identification of a heterozygous splice-site variant, c.4002+1G>A. (C) Direct sequence verification and segregation analysis of *CPAMD8* variants. Variants identified in the probands are indicated by a red dashed box. In individual II:3 of family 3, direct sequencing of *CPAMD8* coding exons that were not covered by WES revealed a heterozygous splice-site variant, c.700+1G>T, in intron 7. The numbering of the cDNA and amino acid residues is in accordance with human *CPAMD8* transcript GenBank: NM_015692.2. Abbreviations are as follows: MAF, minor-allele frequency; NHLBI EVS, National Heart, Lung, and Blood Institute (NHLBI) Exome Sequencing Project Exome Variant Server (EVS); UCL-ex, University College London (UCL) Exomes Consortium; ExAC, Exome Aggregation Consortium; WES, whole-exome sequencing; hemi., hemizygous; het., heterozygous; homo., homozygous; comp. het., compound heterozygous.

Family 1 consisted of an affected South Asian female (II:1), now 24 years old, and her unaffected parents, who are first-cousins (Figure 2A). The proband was initially referred at the age of 8 years with reduced vision and an abnormal eye appearance. There was iris hypoplasia, mild ectropion uveae, nasally displaced pupils with iridodonesis, and posterior cortical cataract (Figures 1A and 1B and Table 1). Ocular examination of the parents was normal.

The unrelated parents in family 2 had no ocular anomalies but had an affected son (white male, II:1), who was first examined at the age of 8 years and is now 17 years of age. The proband was initially referred with mildly reduced vision and abnormal ocular appearances. The irides were displaced nasally and were markedly hypoplastic with ectropion uveae and finger-like extensions from the collarette, suggestive of remnants of the vascularized fetal pupillary membrane. The lens could be visualized through the iris by retroillumination (Figures 1C, 1D, and 1H and Table 1).

Family 3 consisted of two affected white siblings, one male (II:2) and one female (II:3). Their parents are unrelated and had normal ocular examinations. The older brother, II:2, now 53 years old, was first examined at the age of 4 years and found to have normal IOP, iridodonesis, anterior synechiae to Schwalbe’s line, and increased corneal diameters, but without Haab’s striae. The initial diagnosis was arrested congenital glaucoma with buphthalmos, which was revised to megalocornea at the age of 27 years. In spite of a myopic spherical equivalent, he required a reading aid at the age of 32 years. His right crystalline lens became subluxed at the age of 38 years. A right pars planar vitreolensectomy was performed, but he subsequently developed a retinal detachment, which was surgically re-attached. His left lens dislocated into the posterior segment at the age of 41 years and remains in the vitreous cavity, so he is optically aphakic. Both of his pupils are displaced temporally (Figures 1E and 1F and Table 1). His affected sister, II:3, now 50 years old, was first examined at the age of 2 years and found to have normal IOP, increased corneal diameters, thin atrophic irides, and a deeply pigmented angle on the right eye. There were peripheral anterior synechiae involving 90% of the anterior chamber angle bilaterally with a single focal synechial membrane crossing to the angle. She was also diagnosed with arrested congenital glaucoma, which was revised to megalocornea at the age of 25 years. She required reading glasses at the age of 34 years. She developed a right posterior subcapsular lens opacity with superior lens subluxation at the age of 38 years, which was treated by vitreolensectomy and sutured scleral fixation of an intraocular lens (IOL). The IOL dislocated 5 years later and was removed. She then had a left vitreolensectomy at the age of 42 years for nuclear sclerotic cataract and superior lens subluxation. She had a similar iris appearance as her affected brother (II:2), including iris transillumination (Figures 1G and 1H and Table 1). Individual II:2 from family 3 developed ocular hypertension, 26 mmHg (left eye) and 30 mmHg (right eye), at the age of 49 years, and he is on topical treatment. The IOPs of the other individuals are normal (12–18 mmHg), and there was no evidence that any of the other ocular changes have progressed.

To identify the genetic cause(s) of ASD in these families, whole-exome sequencing (WES) was performed on DNA samples from each proband (Figure 2A) with an Agilent SureSelect V5 library preparation kit and HiSeq2000 sequencer (Illumina). Reads were aligned to the human reference sequence (Ensembl Genome browser hg19) with Novoalign version 2.05, and the ANNOVAR tool (OpenBioinformatics) was used to call and annotate sequence variants. WES data were analyzed by ExomeDepth^16^ to identify any potentially causative exonic CNVs. Overall, 25,363, 24,203, and 24,240 exonic sequence alterations were identified in probands from family 1, 2, and 3, respectively (Figure 2B). The average exon sequencing depth was 40×, and 90% of the targeted region was covered with a minimum read depth of 13.

WES variant filtering was performed in four steps, as summarized in Figure 2B. First, on the basis of the hypothesis that ASD-associated mutations are rare, variants with a minor allele frequency (MAF) > 0.005 in the 1000 Genomes database, the National Heart, Lung, and Blood Institute (NHLBI) Exome Sequencing Project Exome Variant Server (EVS), the Exome Aggregation Consortium (ExAC) database, and our internal University College London (UCL) exomes consortium (UCL-ex) database, comprising 1,980 exomes, were filtered (step 1). Rare variants (MAF ≤ 0.005) were then segregated according to their zygosity status (e.g., homozygous, hemizygous, or heterozygous) and cross-referenced with genes previously known to be associated with ASD; no rare variants were identified in any known ASD-associated genes.

Due to the plausible X-linked inheritance of ASD in family 2, an additional step was subsequently performed to analyze X-linked variants (step 2). Only one hemizygous missense variant in *ARMCX4*, c.979A>G (p.Lys327Glu), was identified. However, the lack of potential pathogenicity of this variant, as predicted by the bioinformatic tools (Table S2), suggests that this variant is unlikely to be causative of the condition. In addition, WES data for the proband in family 2 was analyzed by ExomeDepth^16^ to identify any potentially causative exonic CNVs on the X chromosome; no likely deleterious CNVs were identified in any known ASD-associated X-linked genes. CNV analysis of autosomes performed for all three families did not identify any potential exonic CNVs in any autosomal genes associated with ASD.

ASD in family 3 was likely autosomal recessive, whereas in families 1 and 2, either recessive inheritance or a de novo mutation was considered because there were no likely disease-associated X-linked variants for individual II:1 in family 2. Therefore, step 3 selected for plausible candidate autosomal compound heterozygous and homozygous variants in each of the respective families. All variants that remained after filtering step 3 are listed in Table S1. Given the similarity of the ocular features exhibited in all three families, we hypothesized that their condition could be caused by mutations in the same gene. Therefore, in the next filtering step, variants in gene(s) shared by the probands in all three families were selected for further interrogation. This filtering strategy revealed variants in the candidate gene *CPAMD8* (complement 3 and pregnancy zone protein-like, a2-macroglobulin domain-containing protein 8) in probands from families 1 and 2 (Figure 2B). A unique homozygous missense variant, c.4351T>C (p.Ser1451Pro), in *CPAMD8* (GenBank: NM_015692.2) was identified in the proband of family 1, whereas the proband of family 2 carried potential compound heterozygous frameshift, c.2352_2353insC (p.Arg785Glnfs^∗^23), and splice-site, c.4549-1G>A, variants (Figure 2B). However, the stringent filtering only identified compound heterozygous variants in a single gene, *CKAP2*, in the proband of family 3. *CKAP2* was not considered a likely causative gene due to the lack of potential pathogenicity of these variants as predicted by bioinformatic tools (Table S1). This prompted re-analysis of the WES data for the proband of family 3. Upon re-examination of the 116 rare heterozygous variants (MAF ≤ 0.005) after filtering step 1, a unique heterozygous splice-site variant in *CPAMD8*, c.4002+1G>A, was found (Figure 2B). For this sample, WES data lacked exome coverage for six coding exons of *CPAMD8*. The sequencing gaps were then covered by PCR amplification and Sanger sequencing according to standard methodology (primer sequences are available on request). This led to the discovery of a second *CPAMD8* variant in this individual, a unique heterozygous splice-site variant in intron 7, c.700+1G>T (Figure 2C).

**Figure 3:**
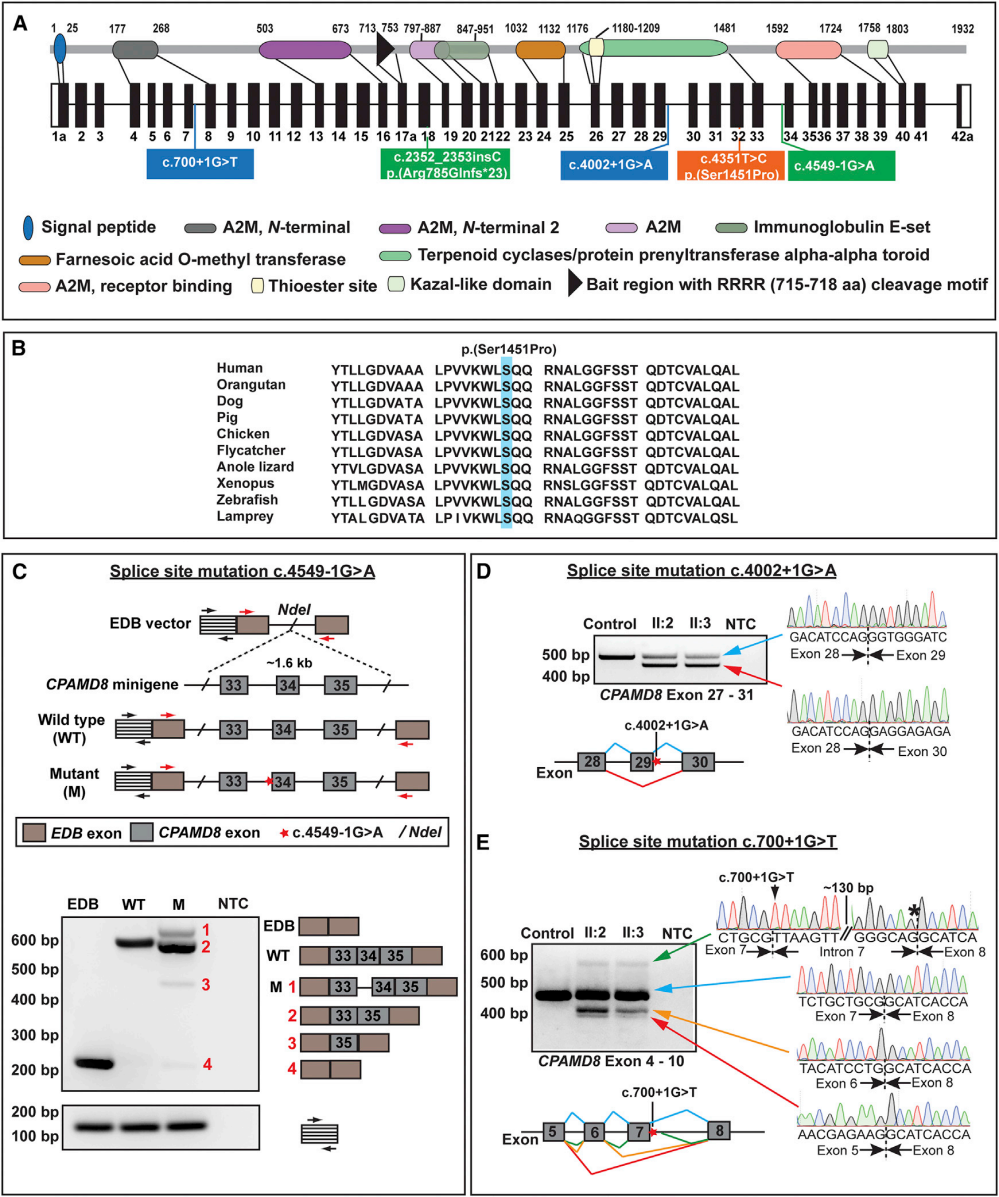
Schematic of CPAMD8 Gene and Protein Structure and Consequence of *CPAMD8* Mutations Identified in this Study (A) Schematic of the genomic and protein structure of CPAMD8-1a. Positions of *CPAMD8* mutations identified in this study are illustrated. The homozygous missense variant, p.Ser1404Pro, identified in family 1 is located in exon 32 (orange box). Compound heterozygous frameshift and splice-site variants, p.Arg785Glnfs^∗^23 and c.4549-1G>A, were identified in family 2 (green boxes). In family 3, compound heterozygous splice-site mutations, c.700+1G>T and c.4002+1G>A, were identified (blue boxes). Mutations are annotated in accordance with transcript *CPAMD8-1a* (GenBank: NM_015692.2). A2M, α2-macroglobulin. (B) Multiple sequence alignment of CPAMD8 orthologs shows that the serine residue altered in family 1, p.Ser1451Pro, is highly conserved in different species. (C) In vitro splice assay of *CPAMD8* via a minigene system. Wild-type and mutant c.4549-1G>A fragments of *CPAMD8* exons 33 to 35 with flanking intronic sequence were cloned into the EDB splice assay vector. The position of the mutation is highlighted with a red star. Primer binding sites to EDB exons are indicated by red arrows, whereas the control primers targeting the vector backbone sequences are depicted by black arrows. Transcripts generated by RT–PCR were separated by agarose gel electrophoresis and were directly sequenced. The wild-type construct generated a correctly spliced *CPAMD8* exon 33-34-35 to the vector exons, whereas the mutant construct generated four aberrantly spliced products. The most efficiently spliced product (2) represents an aberrant splicing event that caused skipping of exon 34. Other aberrantly spliced products detected correspond to the inclusion of intron 33 (1) and the deletion of both exons 33 and 34 (3). The spliced product corresponding to the vector exons alone (4) was also produced by the mutant constructs at a very low level. Amplification with control primers (black arrows) demonstrates equal loading of the cDNA template. NTC, no template control. (D and E) Agarose gel images showing RT-PCR products from whole-blood RNA of affected individuals II:2 and II:3 from family 3, in comparison to a control. Electropherograms and schematic show the transcripts generated by the c.4002+1G>A and c.700+1G>T mutations. Exons are indicated by filled bars and introns are depicted by lines. Splicing events are color coded according to the transcripts in the agarose gel images. (D) Amplification of exons 27–31 encompassing the splice-donor site mutation c.4002+1G>A resulted in two transcripts in both affected siblings. Direct sequencing revealed wild-type product (blue arrow) and a smaller transcript of approximately 470 bp (red arrow), corresponding to exon 29 skipping, which resulted in an in-frame deletion, p.Gly1310_Glu1334del. (E) Wild-type transcript is indicated by a blue arrow. Splice-donor mutation c.700+1G>T resulted in three aberrant transcripts. The top electropherogram (green arrow) shows activation of a cryptic splice-donor site (black asterisk) in intron 7 (142 bp downstream of exon 7), which is predicted to result in a prematurely truncated product, p.Gly234Valfs^∗^30. Two smaller transcripts (orange and red arrows) depict two additional abnormal splicing events, the skipping of exon 7 alone and skipping of exons 6 and 7, which result in frameshifts p.Asp216Alafs^∗^5 and p.Leu210Alafs^∗^5, respectively.

Verification of the disease-associated variants in *CPAMD8* was performed by direct sequencing of the specific exons or introns carrying the variants and segregation analyses in additional family members, when available (primer sequences are available on request). In family 1, each parent was a heterozygous carrier of the missense change, c.4351T>C (p.Ser1451Pro), detected in the proband (II:1) in the homozygous state (Figure 2C). Variant c.4351T>C is absent from 1000 Genomes, NHLBI EVS, our internal UCL-ex database, and the ExAC database (Table 2). This variant is positioned within a highly conserved alpha-2-macroglobulin (A2M) complement component domain of CPAMD8 (Figure 3A), and multiple sequence alignment of CPAMD8 orthologs confirmed that the serine residue is evolutionarily conserved across different species (Figure 3B). Bioinformatic tools SIFT, PolyPhen-2, and PhyloP support the likely pathogenicity of this *CPAMD8* missense variant (Table 2).

In family 2, Sanger sequencing confirmed that the proband (II:1) is compound heterozygous for the c.2352_2353insC (p.Arg785Glnfs^∗^23) and c.4549-1G>A variants, whereas his father (I:1) is heterozygous for the frameshift variant and wild-type for the splice-site variant (Figure 2C). Both c.2352_2353insC and c.4549-1G>A are absent in 1000 Genomes and our internal UCL-ex database and are found at a heterozygous frequency of 21/11,520 alleles and 1/12,356 alleles, respectively, in the NHLBI EVS. In ExAC, the heterozygous frequencies are 47/115,670 alleles for c.2352_2353insC and 1/120,688 alleles for c.4549-1G>A, which is consistent with predicted allele frequencies for a rare recessive disease. No homozygotes for these variants are present in any of the databases (Table 2). Variant c.2352_2353insC is predicted to cause a premature termination, p.Arg785Glnfs^∗^23, whereas variant c.4549-1G>A alters the invariant AG dinucleotide at the splice-acceptor site of intron 33 and is predicted to disrupt normal splicing (Figure 3A).

Given that a fresh blood sample from the family 2 proband was not available, we performed an in vitro splice assay by using a minigene system to test the effect on pre-mRNA splicing, as previously described.^17^, ^18^ Primers were designed to amplify a 1,605 bp *CPAMD8* genomic fragment encompassing exons 33 to 35 and the surrounding intronic regions from the proband’s DNA sample (primer sequences are available on request). PCR products generated were initially cloned into pGEM-T Easy (Promega) and sequenced, then the wild-type and mutant fragments were subcloned into the EDB vector.^17^, ^18^ All constructs generated were directly sequenced to ensure fidelity and orientation (Figure 3C). Wild-type, mutant (c.4549-1G>A), and EDB minigenes were transiently transfected into HEK293 cells with TransIT-LT1 Transfection Reagent (Cambridge BioScience for Mirus Bio). 24 hr after transfection, cells were lysed and homogenized (QIAshredder kit, QIAGEN). Total RNA was extracted via an RNeasy Mini Kit (QIAGEN) with an on-column DNase treatment, according to the manufacturer’s instructions. Approximately 3 μg of total RNA was reverse transcribed to cDNA via the Tetro cDNA Synthesis Kit (Bioline). Reverse transcription (RT)-PCR using EDB vector-specific primers was performed on 100 ng of cDNA with GoTaq Green Master Mix (Promega) (primer sequences are available on request). PCR amplified products were then resolved on an agarose gel (Figure 3C), gel extracted with the QIAquick Gel Extraction Kit (QIAGEN), and directly sequenced with EDB vector-specific primers. Analysis of the resulting transcripts demonstrated that wild-type and mutant constructs produced differently spliced products (Figure 3C). The wild-type construct generated correctly spliced *CPAMD8* exons 33, 34, and 35 to vector exons (Figure 3C), resulting in a transcript of approximately 600 bp. In contrast, four transcripts were produced by the mutant construct. The most abundant product (approximately 550 bp) represents an aberrant splicing event involving exon 34 skipping, resulting in an in-frame deletion of 21 amino acids (aa), p.Ile1517_Gln1537del (Figure 3C). In addition, two aberrantly spliced products corresponding to the inclusion of intron 33, and deletion of exons 33 and 34 were detected and are predicted to produce an in-frame insertion of 26 aa, (p.Ala1516_Ile1517ins26), and an in-frame deletion of 73 aa, (p.Asp1465_Gln1537del), respectively. It is notable that the spliced product corresponding to the vector exons alone (approximately 200 bp) was also produced by the mutant construct at a very low level, suggesting that the splice-site variant reduced the native splicing efficiency within the *CPAMD8* minigene. Thus, our findings demonstrate that the splice-acceptor site variant, c.4549-1G>A caused aberrant pre-mRNA splicing.

In family 3, segregation analysis of two heterozygous splice-site variants, c.4002+1G>A and c.700+1G>T, confirmed that both of the affected siblings (II:2 and II:3) are compound heterozygous for the variants, whereas the unaffected mother (I:2) and unaffected granddaughter (III:1) carry the c.700+1G>T variant only (Figure 2C). Paternal DNA (I:1) was not available for testing. Both c.4002+1G>A and c.700+1G>T variants alter the invariant GT dinucleotide at the splice-donor sites of intron 29 and intron 7, respectively (Figure 3A), and are predicted to abolish the splice-donor sites. To investigate the effects of these variants on pre-mRNA splicing, RT-PCR (primer sequences are available on request) was performed on RNA extracted from lymphocytes from both affected siblings, which is predicted to produce wild-type and aberrant transcripts given that the affected individuals are compound heterozygous for the splice-site variants. Amplification spanning exons 27–31, encompassing the c.4002+1G>A variant, resulted in two transcripts in both affected siblings. Direct sequencing revealed an abundant aberrant transcript (approximately 470 bp) with exon 29 deleted and a low level of wild-type transcript (Figure 3D). The mutation therefore rendered the splice donor site inefficient, with only a residual amount of normal splicing. Deletion of exon 29 in the aberrant transcript is predicted to result in an in-frame deletion of 25 aa, (p. Gly1310_Glu1334del), within the highly conserved A2M complement component domain (Figures 3A and 3D). Amplification of exons 4–10, encompassing the c.700+1G>T variant, revealed several aberrant transcripts and wild-type transcript in both affected siblings. Sequencing revealed a cryptic splice donor site located in intron 7, c.700+142, which is predicted to introduce a premature termination codon 87 bp downstream of exon 7, resulting in a truncated product, p.Gly234Valfs^∗^30. In addition, two smaller transcripts, corresponding to the skipping of exon 7 alone and both exons 6 and 7, were detected that are predicted to produce truncated products, p.Asp216Alafs^∗^5 and p.Leu210Alafs^∗^5, respectively (Figure 3E). In summary, all splice-site mutations identified in families 2 and 3 caused aberrant pre-mRNA splicing, supporting their likely pathogenicity. Notably, the position of mutations identified in this study implies that only isoforms *CPAMD8-1a* and/or *CPAMD8-1b* are important in the pathogenesis of ASD (Figures 3A and S1).

*CPAMD8* is a large gene spanning 134 kb located on chr19p13.11. Investigation of RNA sequencing (RNA-seq) data from the Human BodyMap 2.0 project, human fetal and adult cornea endothelial cells,^19^ basal limbal crypts, superficial limbal crypts,^20^ and whole cornea tissue (unpublished data) using the Integrative Genomics Viewer (IGV) indicated that *CPAMD8* consists of 42 exons that are alternatively spliced into at least two isoforms, *CPAMD8-1a* and *CPAMD8-1b* (Figure S1). *CPAMD8-1a* consists of 42 coding exons, encoding 1,932 aa, and was previously identified in human tissues, including brain, kidney, heart, liver, testis, and small intestine.^21^ Isoform *CPAMD8-1b* uses an alternative acceptor site in intron 41, which gives rise to a different terminal exon 42 (denoted here as exon 42b), resulting in a shorter protein (1,863 aa) (Figure S1). In addition, isoform *CPAMD8-2* was previously identified in human placenta tissue,^22^ although it was absent from the RNA-seq data of other human tissues (Human BodyMap 2.0 project), including ocular tissues.^19^, ^20^ This isoform consists of 14 coding exons, encoding 503 aa. Exon 12 of this isoform is alternatively spliced to exon 17b, which differs from exon 17a by use of an alternative acceptor site in intron 17. This isoform encodes the N-terminal signal peptide and an A2M domain of CPAMD8 (Figure S1).

*CPAMD8* has not previously been implicated in any human disease, but represents an intriguing candidate for recessive ASD because of its potential role in regulating aqueous humor (AH)^23^ and ocular development in semiaquatic amphibians.^24^, ^25^ By comparing the transcriptomes of the choroid plexus epithelium (CPE) in the human brain and the non-pigmented epithelium (NPE) of the ciliary body, *CPAMD8* was found to have significantly higher expression in the NPE than in the CPE.^23^ The NPE produces AH in the eye, which provides nutrients to the eye structures and removes the metabolic waste from the anterior chamber through the trabecular meshwork and Schlemm’s canal.^26^ Interestingly, other genes that are important for the development of the anterior segment, such as *RAX* (MIM: 601881), mutations in which are known to cause microphthalmia (MIM: 611038), also showed higher expression in the NPE. These data suggest an as yet undefined role for *CPAMD8* in the anterior segment of the eye.^23^ Further support for a role for *CPAMD8* in the development of the anterior segment comes from studies in the newt, which is capable of lens regeneration from the dorsal iris pigmented epithelial cells via trans-differentiation. In a study that used RNA-seq to compare differentially expressed genes in regeneration-competent dorsal iris and regeneration-incompetent ventral iris in the newt, *CPAMD8* was found to be upregulated in the regeneration-incompetent ventral iris.^24^ Similarly, microarray analysis performed in another study to identify differentially expressed genes in the iris of young and aged axolotls demonstrated that *CPAMD8* was upregulated in the regeneration-incompetent iris.^25^ Interestingly, *CHRDL1*, which is associated with X-linked megalocornea (MGC1) and adult-onset iris anomalies,^12^, ^27^ was also found to be upregulated in the regeneration-incompetent iris in both amphibians.^24^, ^25^ Together, these findings support an as yet undefined role for *CPAMD8* in the developing anterior segment which, when mutated, might perturb the normal developmental pathway for the anterior segment structures, thereby leading to ASD.

**Figure 4:**
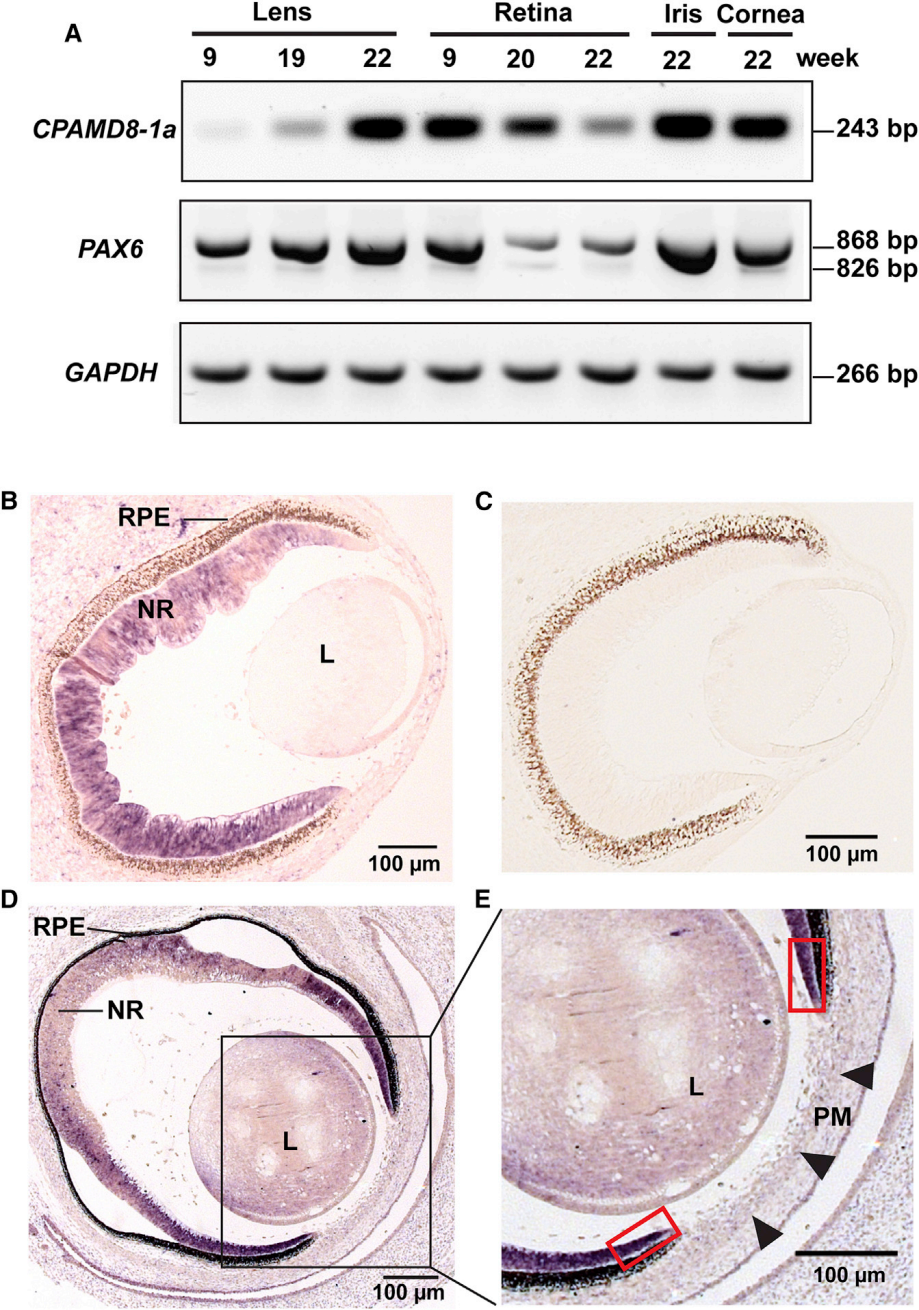
*CPAMD8* Is Expressed in the Developing Human Anterior Segment (A) RT-PCR of human eye tissues showing *CPAMD8-1a* expression in the developing human lens, retina, iris, and cornea with differential temporal expression detected in lens and retina. In the lens, expression of *CPAMD8-1a* increased from the early developmental stage (week 9 of gestation) to the second trimester (week 22 of gestation), whereas in the retina, *CPAMD8-1a* expression gradually declined from early (week 9 of gestation) to later (week 22 of gestation) fetal stages. *PAX6* and *GAPDH* were used as controls. The size of each amplified product is shown. (B–E) In situ hybridization (ISH) of *CPAMD8* in the human eye at Carnegie stage 19 (CS19) (head-sagittal sections) (B and C) and CS23 (face-sagittal sections) (D and E). (B) ISH image shows that *CPAMD8* is strongly expressed in the neural retina (NR) and weakly expressed in the lens (L) at CS19. (C) Sense probe control. (D and E) At CS23, strong *CPAMD8* expression was detected in the distal tips of the retinal neuroepithelium (red boxes). *CPAMD8* expression was weak in the periocular mesenchyme anterior to the lens (L) (arrowheads) at this stage. Abbreviations are as follows: PM, pupillary membrane; RPE, retinal pigmented epithelium.

To explore this hypothesis, we examined the expression pattern of *CPAMD8* in the developing human fetal eye to delineate spatiotemporal expression in the ocular structures implicated in this unique form of ASD. RNA was extracted from microdissected human fetal lens, iris, retina, and cornea with mirVana isolation kits (Thermo Fisher Scientific). First-strand cDNA synthesis was performed with 1 μg total RNA via M-MLV Reverse Transcriptase (Promega) and random hexamer oligonucleotide primers (Invitrogen). Second-strand synthesis was performed with gene-specific primers. Mutations in the C-terminal exons are predicted to affect isoforms *CPAMD8-1a* and *CPAMD8-1b*, implying that only these isoforms are important in the pathogenesis of ASD (Figures 3A and S1). Therefore, we investigated the expression of *CPAMD8-1a* and *CPAMD8-1b* by using RT-PCR with isoform-specific primers (primer sequences are available on request). *CPAMD8*-*1a*-specific primers amplified exon 41 to exon 42 (primer pair 1), whereas *CPAMD8-1b*-specific primers targeted exon 42b (primer pair 2) (Figure S1). *PAX6* and *GAPDH* were included as controls as previously described.^28^ *CPAMD8-1a* expression was detected in the developing human lens and retina as early as week 9 of gestation, and expression was retained into the second trimester (Figure 4A). Expression of *CPAMD8-1a* was also detected in the iris and cornea at week 22 of gestation. Our data demonstrated differential temporal expression of *CPAMD8-1a* in the lens and retina, with increasing levels of expression in the lens from early (week 9 of gestation) to later (week 22 of gestation) developmental stages and decreasing levels of expression in the retina (Figure 4A). *CPAMD8-1b* showed an identical expression pattern to *CPAMD8-1a* in this RT-PCR assay (data not shown).

To further explore the expression of *CPAMD8*, we performed in situ hybridization (ISH) in the developing human embryonic eye. Two riboprobes were synthesized with Digoxigenin-UTP RNA labeling kits (Roche) from a fragment of 356 bp amplified from the *CPAMD8* 3′ UTR in genomic DNA (probe A) or a 392 bp fragment of *CPAMD8* exon 4 to 10 amplified from lymphoblast-derived cDNA (probe B; Figure S1) (primer sequences are available on request). These riboprobes were designed to target both isoforms *CPAMD8-1a* and *CPAMD8-1b* (Figure S1) and were cloned into pGEM-T Easy (Promega). Human embryonic eyes were fixed in 4% (w/v) phosphate-buffered paraformaldehyde solution and embedded in paraffin wax before sectioning. ISH was performed in 300 mM NaCl, 5 mM EDTA, 20 mM Tris-HCl, 5 mM sodium phosphate, 0.1 mg/mL yeast tRNA, 10% dextran sulfate, 1× Denhardt’s reagent, 0.5 mg/mL tRNA, and 50% formamide with digoxigenin-incorporated riboprobes at 65°C. Post-hybridization slides were incubated with anti-digoxigenin conjugated with alkaline phosphatase (Roche) diluted 1:1,000 in 2% fetal calf serum. Expression patterns were visualized with a Nitro-Blue Tetrazolium Chloride/5-Bromo-4-Chloro-3-Indolyphosphate p-Toluidine Salt (NBT/BCIP) system (Roche). Sections were mounted with Vectamount (Vector laboratories) and analyzed with a Zeiss Axioplan 2 imaging system. Embryonic eyes at Carnegie stages (CS) 18 (day 44), 19 (day 47–48), 21 (day 52) and 23 (day 56–57) were analyzed. Both antisense riboprobes produced the same results, and data from probe A is shown.

Consistent with the RT-PCR findings, ISH results revealed robust *CPAMD8* expression in the developing embryonic neural retina from the seventh to the eighth week, whereas lower expression was detected in the embryonic lens (Figures 4A and 4B). Notably, ISH revealed strong expression of *CPAMD8* in the distal tips of the retinal neuroepithelium that contributes to the development of the iris and ciliary body by the eighth week (Figure 4D and 4E). Interestingly, ISH did not detect significant embryonic expression of *CPAMD8* in the periocular mesenchyme, or in the mesenchyme anterior to the lens, corresponding to the pupillary membrane and developing cornea at CS 23 (Figure 4E), whereas *CPAMD8* expression was readily detected by RT-PCR at later stages in the fetal lens, iris, and cornea. The pupillary membrane contributes to the development of iris stroma and will degenerate during the eighth month of gestation. Thus, the *CPAMD8* expression profile in the developing anterior segment of the embryonic and fetal eye correlates with the affected tissues and ocular phenotype, characterized by predominant iris and lens anomalies with the appearance of possible persistent pupillary membrane remnants in one affected individual. Our data imply that impaired *CPAMD8* function disrupts the normal development of the lens and iris structures. Signaling from the lens during development has been shown to have an important role in forming the structures of the anterior segment,^29^, ^30^ suggesting that the perturbed *CPAMD8* function in the developing lens could be another contributory factor in the maldevelopment of the iris in these individuals.

*CPAMD8* is a member of the A2M/C3 (alpha-2-macroglobulin/complement 3) protein family,^21^ which is comprised of six other members, including A2M,^31^ PZP (pregnancy zone protein),^32^ CD109,^33^ and complement proteins C3,^34^ C4,^35^ and C5.^36^ The proteins in the A2M/C3 family play different roles in the innate and acquired immune system. A2M is a soluble versatile proteinase inhibitor, and interaction with growth factors, such as TGF-β (transforming growth factor-β), has been described.^37^, ^38^ A2M inhibits protease activity through a unique mechanism. Upon binding to the protease, it triggers the proteolytic cleavage of A2M in its bait region, which causes A2M to entrap the protease, followed by the removal of the complex by the LDL (low density lipoprotein) receptor family.^37^, ^38^, ^39^, ^40^ Similar to A2M, PZP is a soluble protease inhibitor, although a different protease inhibition mechanism has been reported.^41^ A high amount of PZP is present in the serum during pregnancy.^32^ Complement proteins, which are also soluble, play an important role in defending against pathogens.^42^ In contrast to other A2M/C3 family members, CD109 is a membrane-bound glycosylphosphatidylinositol (GPI)-anchored protein^33^ and has been shown to negatively modulate TGF-β1 in human keratocytes.^43^ All A2M/C3 members, except C5, have a characteristic thioester (TE) bond, which enables the protein to bind covalently to the target protease. Consistent with other members of the A2M/C3 family, CPAMD8 has an N-terminal signal peptide, a TE motif, A2M-associated domains, and a C-terminal Kazal-like domain. Interestingly, Kazal-domain proteins, such as FSTL3 (follistatin-related protein 3), have been shown to act as antagonists for TGF-β family members, which negatively regulates the BMP signaling pathway.^44^, ^45^ A similar function has been described for CHRDL1, which is associated with MGC1.^12^ In addition to a Kazal-like domain in the C terminus, CPAMD8 has an RRRR conserved site at residues 715–718, which are features of complement proteins^21^ (Figure S2). The function of CPAMD8 is unknown, but membrane association and proteolytic processing into two chains of approximately 70 and 130 kDa has been demonstrated.^21^

**Figure 5:**
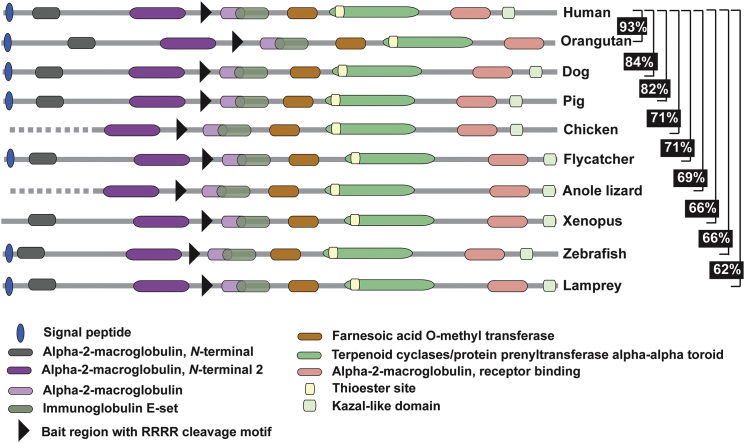
Schematic Representation of CPAMD8 Orthologs Showing Highly Conserved Functional Motifs from Human to Lamprey All CPAMD8 orthologs contain the hallmark motifs of the A2M/C3 family, including an N-terminal signal peptide, bait region with cleavage site, and thioester site. CPAMD8 has the alpla-2-macroglobulin specificity-determining domains, also found in A2M. CPAMD8 also has a conserved RRRR processing site that is found in complement proteins, and a unique C-terminal domain, which in CPAMD8 is a Kazal-like domain. The pairwise full-length protein sequence identities between human and other species are indicated, ranging from 62%–93% sequence identity. Dotted lines indicate incomplete sequences.

**Figure 6:**
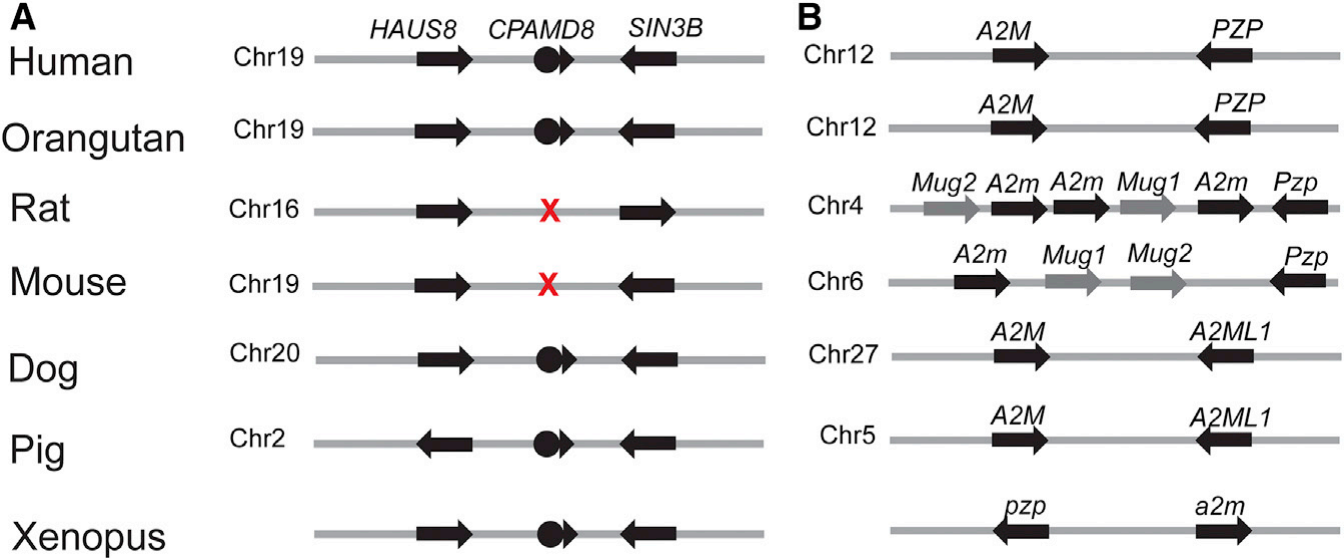
Synteny of Gene Clusters for *CPAMD8*, *A2M*, *PZP*, *Mug1*, *and Mug2* in Different Species (A) Schematic depicting *CPAMD8* flanked by *HAUS8* and *SIN3B* in different species. X indicates deletion of *CPAMD8* from the syntenic region in rat and mouse genomes. Flanking genes are shown as arrows. (B) Schematic showing the clustered location of *A2M* and *PZP* in different species and *Mug1* and *Mug2* on the same chromosome in rodents. The chromosomal position of *Mug1* and *Mug*2 adjacent to *A2m* and *Pzp* suggests that they are a result of tandem duplication. The distance of the genes depicted is not to scale. Gene orientation is indicated by the arrowhead.

The emergence of the A2M/C3 gene family took place early in evolution. It is believed that the A2M/C3 family of genes are the result of duplication from a common ancestral gene that then distributed to different chromosomes.^46^ This is supported by the clustered distribution of A2M/C3 family of genes in different species. For example, in humans, *CPAMD8* and *C3* are clustered on chromosomal region 19p13.1–13.3, 12p13.3 carries *A2M* and *PZP*, and *C4* and *CD109* are found on 6p21.3 and 6q13, respectively. However, the A2M/C3 gene family has undergone divergence in different species.^46^ For example, *A2M* and *C4* are duplicated in zebrafish^46^ and mice.^47^ Given that A2M/C3 family members play an important role in innate and acquired immunity, differential evolution of the A2M/C3 family proteins could represent the requirement for distinctive defense mechanisms in different species. Interestingly, although CPAMD8 is highly conserved in different species, from humans to jawless fish, with protein sequence identity ranging from 62%–93% (Figure 5), we show that CPAMD8 was lost in the rodent lineage (Figure 6A). Instead, rodents have a different clustered family member, murinoglobulins, including Mug1 and Mug2, which are absent in humans (Figure 6B). Mug1 and Mug2 are highly homologous, sharing approximately 90% sequence identity. Comparison of the protein sequence of mouse Mug1 and Mug2 to human A2M/C3 members revealed that Mug1 and Mug2 share higher sequence identities with A2M (59%) and PZP (57%) than with the other family proteins, CD109 (33%), C4 (27%), C3 (25%), and C5 (23%). The conserved motifs in mouse Mug1 and Mug2 and other human A2M/C3 family members are shown in Figure S2. Furthermore, their clustered location with *A2m* and *Pzp* on mouse chromosome 6 and rat chromosome 4 suggests that *Mug1* and *Mug2* are products of tandem duplication of the *A2m/Pzp* protogene (Figure 6B). Moreover, mouse Mug1 and Mug2 have <33% homology to human CPAMD8, demonstrating that Mug1 and Mug2 are not mouse orthologs of CPAMD8, but most likely represent paralogs in the protein family. This lineage-specific deletion of *CPAMD8* and the exclusive introduction of murinoglobulins in rodents reflect genetic buffering or functional divergence (existence of alternative pathways) between humans and rodents. Given the conserved functional motifs shared among all A2M/C3 family members, it is likely that the lack of requirement of *CPAMD8* in rodents results from the presence of redundant or backup genes within the family, which provide functional complementation. In addition, it is possible that the absence of *CPAMD8* in rodents re-routes the task to an alternative pathway, which would render *CPAMD8* redundant in rodents. Both hypotheses suggest a more complicated underlying functional complementation or divergence between the human and rodent lineage. Future studies will be required to fully elucidate this lineage-specific redundancy.

Our study has several important implications for understanding the etiology of ASD and the development of the anterior segment. First, we define a unique form of autosomal-recessive ASD, characterized by predominant iris and lens anomalies that don’t affect the cornea or posterior segment. Second, we identify mutations in *CPAMD8*, encoding a protein of unknown function, as the cause of autosomal-recessive ASD. Third, our data demonstrate the spatiotemporal expression of *CPAMD8* in the developing human lens, iris, retinal neuroepithelium, and presumptive cornea, suggesting that *CPAMD8* might have a potential role in crosstalk between the optic cup peripheral neuroepithelium and the anterior periocular mesenchyme during eye morphogenesis. Finally, phylogenetic analyses show the rodent lineage-specific deletion of *CPAMD8*, implying functional divergence or complementation of gene function between humans and rodents. In conclusion, our study delineates a clinical entity, and our findings suggest that mutations in *CPAMD8* might cause ASD in genetically unresolved individuals.

## Figures and Tables

**Table 1:** Clinical Details of the Four Individuals Identified with *CPAMD8* Mutations

	**Family 1**		**Family 2**		**Family 3**			
	**II:1**		**II:1**		**II:2**		**II:3**	
Gender	female		male		male		female	
Ethnicity	south Asian		white		white		white	
Age (age at last follow up)	24 years (NA)		17 years (NA)		53 years (53)		50 years (44)	

	**R Eye**	**L Eye**	**R Eye**	**L Eye**	**R Eye**	**L Eye**	**R Eye**	**L Eye**

HWTW (mm)	12	12	NA	NA	13	13.5	13.5	13.5
Visual acuity	6/12	6/24	6/6	6/9.5	6/24	6/6	6/6	6/9
Refraction before surgery	−2.00/−2.00 × 155	−4.00/−3.50 × 15	NA	NA	−5.25/−2.00 × 117	−3.75/−1.75 × 78	−0.50/−1.25 × 35	−0.5/−1.50 × 150
Cataract (type)	yes (posterior cortical)	yes (posterior cortical)	yes (posterior cortical)	yes (posterior cortical)	yes (NA)	yes (NA)	yes (posterior subcapsular)	yes (nuclear sclerotic)
Cataract surgery (month/date/year)	no	no	no	no	yes (5/24/2002)	no	yes (10/3/2002)	yes (7/21/2008)
Ectopia lentis	NA	NA	nasal	nasal	yes	yes	yes	yes
Microphakia	NA	NA	yes	yes	no	no	no	no
Corectopia	nasal	nasal	nasal	nasal	inferotemporal	inferotemporal	inferotemporal	inferotemporal
Ectropion uveae	yes	yes	yes	yes	yes	yes	no	no
Iris hypoplasia	yes	yes	yes	yes	no	no	yes	yes
Iris transillumination	no	no	yes	yes	yes	yes	yes	yes
Iridodonesis	yes	yes	yes	yes	yes	yes	no	no
Iridocorneal angle	normal	normal	normal	normal	adhesions^a^	adhesions^a^	adhesions^a^	adhesions^a^
Foveal hypoplasia	NA	NA	NA	NA	no	no	no	no
Optic nerve dysplasia	yes	yes	NA	NA	no	no	no	no
Disc (cup:disc)	0.6	0.6	NA	NA	0.3	0.4	0.4	0.4
IOP (mmHg)	18	18	12	12	30	26	17	17
Descemet’s membrane split	no	no	no	no	no	no	no	no
Corneal opacity	no	no	no	no	no	no	no	no
Corneal edema	no	no	no	no	no	no	NA	NA
Retinal detachment (month/date/year)	no	no	no	no	10/14/2002	no	no	no
Other ocular phenotype	hypertrophic collarette, right convergent squint, horizontal nystagmus (both eyes)		persistent pupillary membrane	persistent pupillary membrane	–	–	–	anterior capsule pigmentation
Facial dysmorphism	NA		pinched nostrils (parents do not share this feature)		no		no	

Abbreviations are as follows: R, right; L, left; NA, not available; HWTW, cornea horizontal diameter (white to white); IOP, intraocular pressure.

a Adhesions to Schwalbe’s line.

**Table 2:** Summary of Autosomal-Recessive ASD *CPAMD8* Mutations

**Family**	**Exon or Intron**	**Nucleotide Change**	**Protein Change**	**PolyPhen-2 (Human Variation Score 0–1)**	**SIFT (Tolerance Index 0–1)**	**PhyloP**	**UCL-ex (Exomes)**	**1000 Genomes**	**NHLBI EVS Total Alleles**	**ExAC Total Alleles**	
										**Het.**	**Homo.**
1	exon 32	c.4351T>C	p.Ser1451Pro	PRD (0.948)	damaging (0.01)	conserved (0.98)	0/1,980	0	0/12,378	0/116,178	0/116,178
2	exon 18	c.2352_2353insC	p.Arg785Glnfs^∗^23	NA	NA	NA	0/1,980	0	21/11,520	47/115,670	0/115,670
	intron 33	c.4549–1G>A	p.?	NA	NA	NA	0/1,980	0	1/12,356	1/120,688	0/120,688
3	intron 7	c.700+1G>T	p.?	NA	NA	NA	0/1,980	0	0/12,140	0/120,212	0/120,212
	intron 29	c.4002+1G>A	p.?	NA	NA	NA	0/1,980	0	0/12,350	0/120,350	0/120,350

In silico analysis of rare *CPAMD8* variants identified. PolyPhen-2 appraises mutations quantitatively as benign, possibly damaging, or probably damaging (PRD) on the basis of the model’s false-positive ratio. SIFT results are reported to be tolerant if tolerance index is ≥0.05 or intolerant if tolerance index is <0.05. PhyloP prediction is conserved if score is >0.95, otherwise non-conserved. The cDNA is numbered according to GenBank: NM_015692.2. Abbreviations are as follows: NA, not available; UCL-ex, University College London (UCL) exomes consortium; NHLBI EVS, National Heart, Lung, and Blood Institute (NHLBI) Exome Sequencing Project Exome Variant Server (EVS); ExAC, Exome Aggregation Consortium; Het., heterozygote; Homo., homozygote.

## Web Resources

1000 Genomes, http://browser.1000genomes.org/index.html
ANNOVAR, http://annovar.openbioinformatics.org/en/latest/
BLAST, http://blast.ncbi.nlm.nih.gov/Blast.cgi
Ensembl Genome Browser, http://www.ensembl.org/index.html
ExAC Browser, http://exac.broadinstitute.org/
ExPASy, http://web.expasy.org/translate/
GenBank, http://www.ncbi.nlm.nih.gov/genbank/
Interactive Genomics Viewer (IGV), https://www.broadinstitute.org/software/igv/
NHLBI Exome Sequencing Project (ESP) Exome Variant Server, http://evs.gs.washington.edu/EVS/
Novocraft, http://www.novocraft.com
OMIM, http://www.omim.org/
PolyPhen-2, http://genetics.bwh.harvard.edu/pph2/
SIFT, http://sift.jcvi.org/
UniProt, http://www.uniprot.org/

## Supplemental Data

Document S1. Figures S1 and S2 and Tables S1 and S2

Document S2. Article plus Supplemental Data
