# Supplementary material for: Mutations in CPAMD8 Cause a Unique Form of Autosomal-Recessive Anterior Segment Dysgenesis
Source: Am J Hum Genet. 2016 Nov 10;99(6):1338–52. doi: 10.1016/j.ajhg.2016.09.022 (PMC5142107; doi:10.1016/j.ajhg.2016.09.022)
Supplement: Document S1. Figures S1 and S2 and Tables S1 and S2 [file mmc1.pdf]

The American Journal of Human Genetics, Volume *99*

Supplemental Data

Mutations in *CPAMD8* Cause a Unique Form of Autosomal-Recessive Anterior Segment Dysgenesis

Sek-Shir Cheong, Lisa Hentschel, Alice E. Davidson, Dianne Gerrelli, Rebecca Davie, Roberta Rizzo, Nikolas Pontikos, Vincent Plagnol, Anthony T. Moore, Jane C. Sowden, Michel Michaelides, Martin Snead, Stephen J. Tuft, and Alison J. Hardcastle

SUPPLEMENTAL DATA

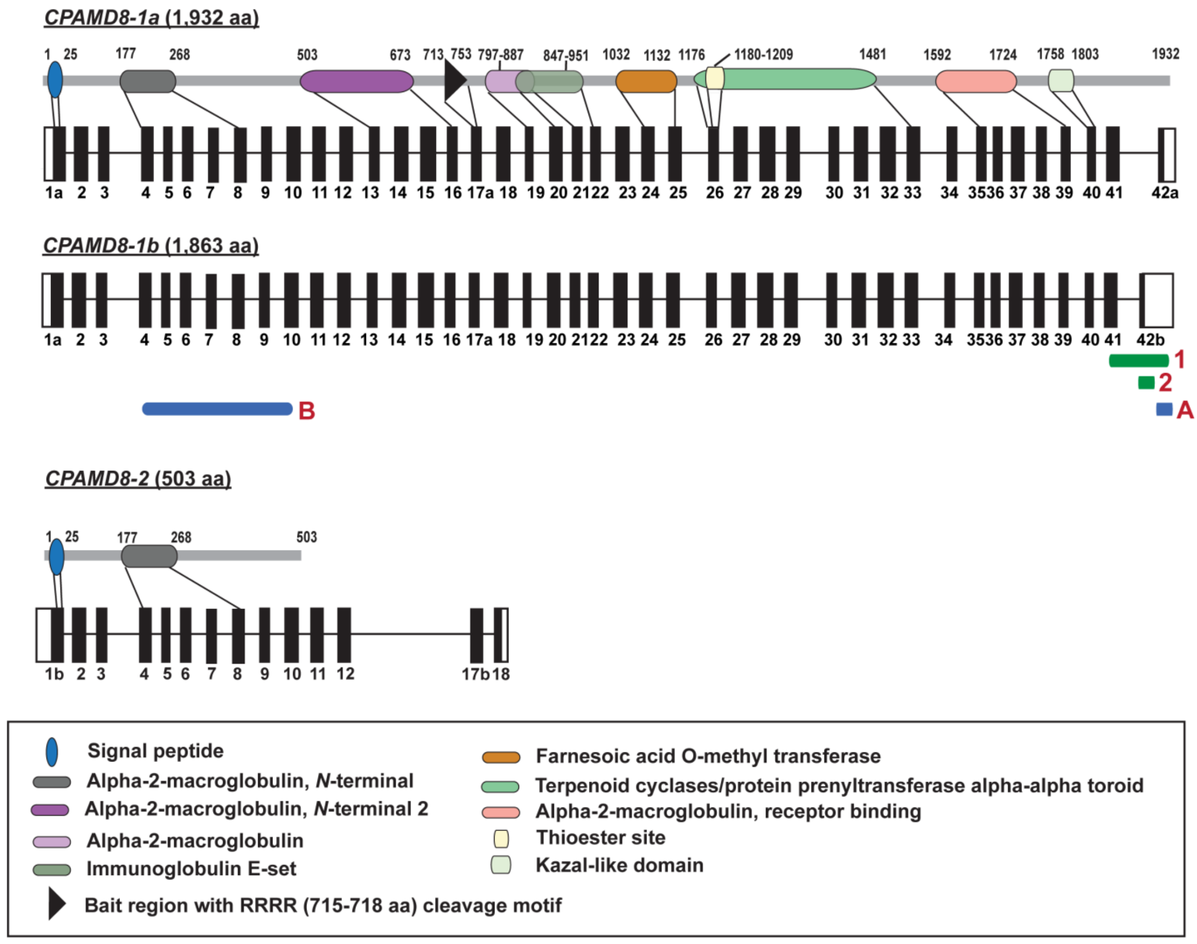

Figure S1. Schematic of genomic and protein structure of CPAMD8 isoforms and position of RT-PCR primers and riboprobes for *in situ* hybridization. Isoforms *CPAMD8-1a* and *CPAMD8-1b* both consist of 42 coding exons, with an alternative terminal exon 42. *CPAMD8-1a* encodes 1,932 amino acids, whereas isoform *CPAMD8-1b* encodes 1,863 amino acids. Isoform *CPAMD8*-2 consists of 14 coding exons, which encode 503 amino acids. The protein encoded by *CPAMD8-2* lacks *C*-terminal domains. Products of RT-PCR primer pairs are indicated by green bars (not to scale). Primer pair 1, *CPAMD8-1a*-specific; Primer pair 2, *CPAMD8-1b*-specific. Blue bars (not to scale) labeled with A and B indicate the riboprobes for *in situ* hybridization. Probe A targets the 3’ untranslated region of *CPAMD8*, whereas probe B binds to the *CPAMD8* exons 4 to10.

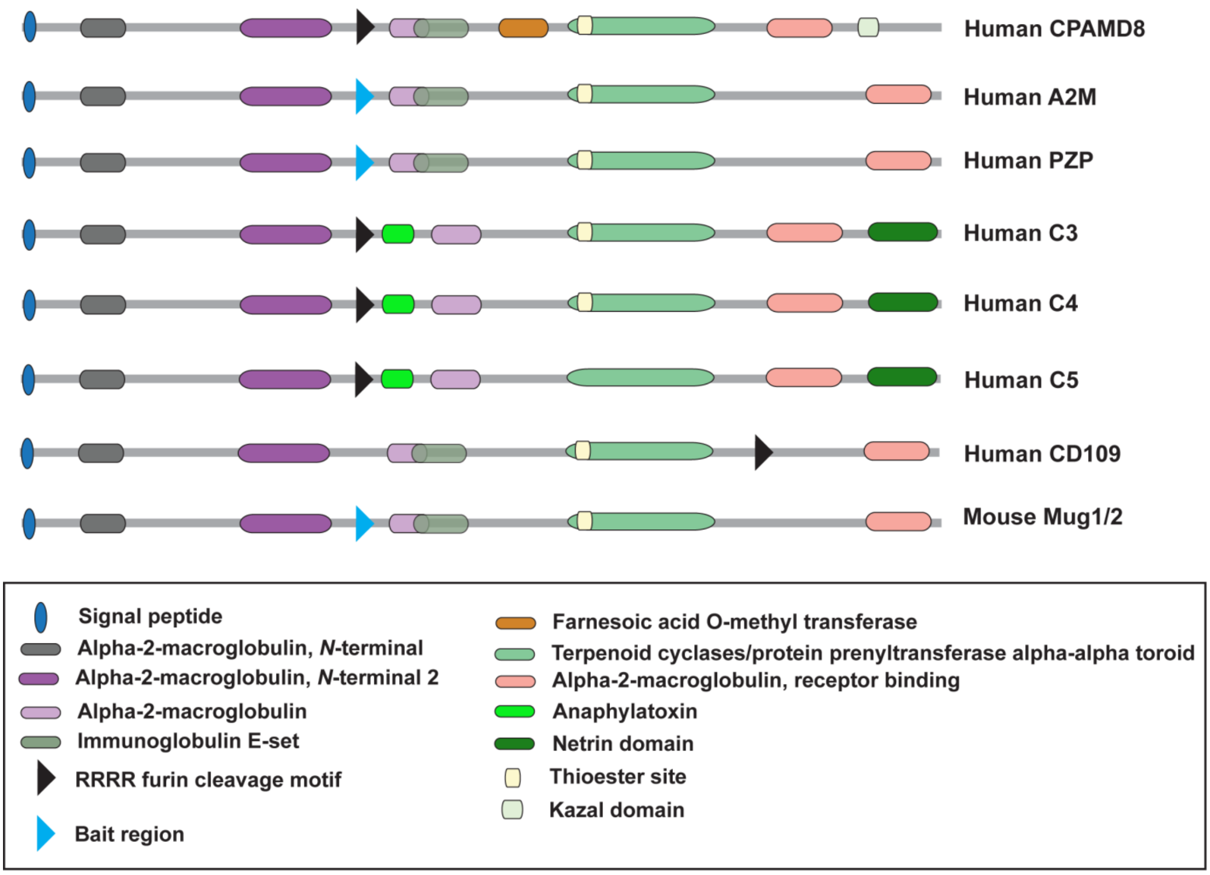

Figure S2. Schematic representation of conserved motifs in A2M/C3 family members compared to human CPAMD8. All A2M/C3 family proteins consist of an *N*-terminal signal peptide, bait region or furin cleavage motif, and a thioester (TE) site, except C5, which is lacking a TE binding site. All complement proteins and CPAMD8 have a conserved RRRR processing site, and a unique *C*-terminal domain, which is a netrin in complement proteins, and a Kazal-like domain, in CPAMD8. A2M and PZP have a bait region, which is absent in complement proteins. The furin cleavage motif located after the TE site is only found in CD109. Comparison of mouse Mug1 and Mug2 to the human A2M/C3 family proteins shows that Mug1 and Mug2 share more conserved functional domains with A2M and PZP than to any other family members.

Table S1: Summary of WES variants

Family (Individual)	HUGO gene	Het./Homo.	Nucleotide change	Protein change	Polyphen 2 (human variation score 0-1)	SIFT (tolerance index 0-1)	Blosum62 (-4 to 11)	UCL-ex (individuals)	1000 Genomes	NHLBI EVS total alleles	ExAC total alleles	
											Het.	Homo.
**1 (II:1)**	*POM121L2*	Het.	c.829G>A	p.(Val277Ile)	BNG (0.132)	T (0.15)	3	0/1980	0	0/4,566	0/21,244	0/21,244
		Het.	c.245A>G	p.(Lys82Arg)	BNG (0.078)	T (0.42)	2	0/1980	0	0/4,566	0/21,096	0/21,096
	OTOG	Het.	c.2104C>T	p.(His702Tyr)	PRD (0.934)	T (0.4)	2	0/1980	0	NA	1/14,602	0/14,602
		Het.	c.8011G>A	p.(Val2671Met)	BNG (0.005)	T (0.14)	1	0/1980	0	NA	1/15,334	0/15,334
	DNASE1L2	Het.	c.437T>C	p.(Leu146Pro)	BNG (0.002)	T (0.23)	-3	0/1980	0	0/11,348	0/43,290	0/43,290
		Het.	c.442T>C	p.(Ser148Pro)	BNG (0.001)	T (0.21)	-1	0/1980	0	0/11,348	0/37,574	0/37,574
	GOLGB1	Homo.	c.7906C>T	p.(His2636Tyr)	BNG (0.187)	D (0.03)	2	0/1980	0	0/13,006	11/121,180	0/121,180
	GOLGB1	Homo.	c.6244T>A	p.(Leu2082Ile)	POS (0.904)	T (0.17)	2	0/1980	0	0/13,006	11/120,880	0/120,880
	IQCB1	Homo.	c.1000A>C	p.(Lys334Gln)	PRD (0.977)	T (0.56)	1	0/1980	0	0/13,006	12/121,400	0/121,400
	CASC1	Homo.	c.1936G>A	p.(Val646Ile)	PRD (0.991)	T (0.49)	3	0/1980	0.0009	11/12,994	24/120,408	0/120,408
	CASC1	Homo.	c.1388C>A	p.(Pro463Gln)	BNG (0.190)	T (0.36)	-1	0/1980	0	0/12,994	0/121,378	0/121,378
	ITPR2	Homo.	c.2976G>T	p.(Met992Ile)	BNG (0.006)	T (0.36)	1	0/1980	0	0/11,954	0/120,370	0/120,370
	SLC15A4	Homo.	c.775G>A	p.(Asp259Asn)	BNG (0.028)	T (0.21)	1	0/1980	0	0/13,006	1/121,402	0/121,402
	FARSA	Homo.	c.1072G>A	p.(Glu358Lys)	BNG (0.426)	D (0)	1	0/1980	0	0/13,006	0/121,306	0/121,306
	CPAMD8	**Homo.**	**c.4351T>C**	**p.(Ser1451Pro)**	**PRD (0.948)**	**D (0.01)**	**-1**	**0/1980**	**0**	**0/12,378**	**0/116,178**	**0/116,178**

**2 (II:1)**	*EVX2*	Het.	c.128C>A	p.(Ser43*)	NA	NA	NA	0/1980	0	0/13,006	0/121,190	0/121,190
		Het.	c.127T>G:	p.(Ser43Ala)	BNG (0.001)	T (0.61)	1	0/1980	0	0/13,006	0/121,190	0/121,190
	UTRN	Het.	c.925A>G	p.(Met309Val)	BNG (0.008)	T (0.45)	1	0/1980	0	0/13,006	0/121,386	0/121,386
		Het.	c.2921T>C	p.(Leu974Pro)	PRD (0.987)	D (0)	-3	0/1980	0	3/13,004	3/120,180	0/120,180
	CPAMD8	**Het.**	**c.4549-1G>A**	**p.?**	**NA**	**NA**	**NA**	**0/1980**	**0**	**1/12,356**	**1/120,688**	**0/120,688**
		**Het.**	**c.2352_2353 insC**	**p.(Arg785Glnfs*23)**	**NA**	**NA**	**NA**	**0/1980**	**0**	**21/11,520**	**47/115,670**	**0/115,670**
**3 (II:3)**	*CKAP2*	Het.	c.91A>G	p.(Lys31Glu)	BNG (0.009)	T (1)	1	0/1980	0	0/13,004	2/121,264	0/121,264
		Het.	c.1748C>T	p.(Thr583Met)	BNG (0.128)	D (0.01)	-1	0/1980	0	0/13,004	2/121,006	0/121,006

Abbreviations are as follow: UCL-ex, University College London (UCL) exomes consortium; NHLBI EVS, National Heart, Lung, and Blood Institute (NHLBI) Exome Sequencing Project Exome Variant Server (EVS); ExAC, Exome Aggregation Consortium; Het., heterozygous; Homo., homozygous. *In silico* analysis of rare variants identified is presented. Polyphen 2 appraises mutations quantitatively as benign (BNG), possibly damaging (POS) or probably damaging (PRD) based on the model's false positive ratio. SIFT results are reported to be tolerant (T) if tolerance index is ≥0.05 or damaging (D) if tolerance index is <0.05. Blosum62 substitution matrix score positive numbers indicate a substitution more likely to be tolerated evolutionarily and negative numbers suggest the opposite. *CPAMD8* mutations identified in this study are highlighted in bold. The cDNA is numbered according to the following RefSeq transcript ID, *POM121L2* (NM_033482.3), *OTOG* (NM_001277269.1), *DNASE1L2* (NM_001374.2), *GOLGB1* (NM_001256486.1), *IQCB1* (NM_001023570.3), *CASC1* (NM_001082972.2), *ITPR2* (NM_002223.3), *SLC15A4* (NM_145648.3), *FARSA* (NM_004461.2), *EVX2* (NM_001080458.1), *UTRN* (NM_007124.2), *CKAP2* (NM_001098525.2), and *CPAMD8* (NM_015692.2).

Table S2: X-linked variant identified in the proband from family 2

HUGO gene	Het./Homo./Hemi.	Nucleotide change	Protein change	Polyphen 2 (human variation score 0-1)	SIFT (tolerance index 0-1)	Blosum62 (-4 to 11)	UCL-ex (individuals)	1000 Genomes	NHLBI EVS total alleles	ExAC total alleles	
										Het.	Homo.
ARMCX4	Hemi.	c.979A>G	p.(Lys327Glu)	POS (0.807)	T (1.00)	1	0/1980	0	NA	1/3,000	0/3,000

Abbreviations are as follow: UCL-ex, University College London (UCL) exomes consortium; NHLBI EVS, National Heart, Lung, and Blood Institute (NHLBI) Exome Sequencing Project Exome Variant Server (EVS); ExAC, Exome Aggregation Consortium; Hemi., hemizygous. *In silico* analysis of *ARMCX4* variant identified is presented. Polyphen 2 appraises mutations quantitatively as benign, possibly damaging (POS) or probably damaging based on the model's false positive ratio. SIFT results are reported to be tolerant (T) if tolerance index is ≥0.05 or damaging if tolerance index is <0.05. Blosum62 substitution matrix score positive numbers indicate a substitution more likely to be tolerated evolutionarily and negative numbers suggest the opposite. The cDNA is numbered according to the RefSeq transcript ID NM_001256155.2.
